# The complete plastid genome and phylogenetic analysis of *Codium fragile*

**DOI:** 10.1080/23802359.2019.1704196

**Published:** 2020-01-07

**Authors:** Xuli Jia, Tao Liu, Ruoran Li, Yuemei Jin

**Affiliations:** a Laboratory of Genetics and Breeding of Marine Organism, College of Marine Life Sciences, Ocean University of China, Qingdao, China;; b College of Life Sciences, Yantai University, Yantai, China

**Keywords:** *Codium fragile*, plastid genome, phylogenetic analysis, Chlorophyta

## Abstract

*Codium fragile*, a nutrient-rich green algae that is both edible and medicinal, it is called a ‘rat tail’ because of its unique shape, it can reach up to a metre in length and has the functions of clearing heat and detoxifying, detumescence and water, and repelling insects. The plastid genome sequence of *C. fragile* is 83,422 bp. A total of 105 genes were determined, including 77 protein-encoding genes, 3 rRNA genes, and 25 tRNA genes. Phylogenetic analysis showed that *C. fragile* clustered together into a single branch with *C. simulans* and *Codium* sp. *'arenicola'* as sister branches. The plastid genome analysis will help the understanding of Chlorophyta evolution.

*Codium fragile* is a popular, edible and medicinal green alga belonging to the family Codiaceae. It is widely distributed along the shores of East Asia, Oceania and Northern Europe (Chaiwat Monmai et al. 2019). It has also been used as an oriental medicine for the treatment of diseases, such as enterobiosis, dropsy, and dysuria (Lee et al. 2010). *Codium fragile* extracts have been demonstrated to possess thrombolytic, anticoagulant, antiplatelet (Choi et al. 2013), anti-inflammatory (Kang et al. 2012). However, genomic studies on this species are relatively limited. In this study, we sequenced, embeled and annotated the plastid DNA of *C. fragile,* conducted a systematic genome study to evaluate the evolutionary trend of plastid DNA, and studied the phylogenetic relationship between *C. fragile* and other green algae with red algae *Gracilaria firma* (NC_033877.1) served as the out-group.

The determination of the complete *C. fragile* plastid genome sequence by next-generation sequencing methods was conducted. The specimen was collected from China (Rongcheng, Shandong Province, 37°08′26.07″N, 122°26′45.39″E) and stored at the Culture Collection of Seaweed at the Ocean University of China (accession no. 2016070047). The total DNA was extracted using the modified CTAB method (Doyle and Doyle 1990). Paired-end reads (150 bp) were sequenced by using Illumina HiSeq system (Illumina, San Diego, CA). tRNAscan-SE Search Server (Schattner et al. 2005) were used to identified the tRNA genes. The other plastid genomic regions were annotated with Geneious R10 (Biomatters Ltd, Auckland, New Zealand), using the *Codium* sp. *'arenicola'* (NC_037366.1) and *C. simulans* (NC_032043.1) plastid genome as reference sequences.

The complete *C. fragile* plastid genome is a circular DNA molecule measuring 83,422 bp in length with the overall G + C content of the complete plastid genome is 27.3% (GenBank accession no. MN733705). The plastid genome contains 105 genes, including 77 protein-coding genes, 3 rRNA genes (*rnl* rRNA, *rns* rRNA and *rrn5* rRNA) and 25 tRNA genes. The nucleotide composition was 35.56% A, 12.93% C, 14.34% G, and 37.17% T. The length of the coding region was 70,700 bp, corresponding to 84.75% of the total length. Of the 77 protein-coding genes in *C. fragile,* 66 (85.71%) started with the initiation codon ATG and 11 (14.29%) started with the non-initiation codon ATG, 68 (88.31%) ended with the TAA stop codon, 4 (5.19%) with TAG (*ycf12*, *cemA, chlB* and *psbZ*) and 5 (6.49%) with TGA (*chlL*, *atpI*, *rps2, rpl32* and *ycf47*).

Twenty-three shared plastid genome protein sequences from 17 green algae and 1 red algae including *Codium fragile* were used to conduct phylogenetic analysis by using MrBayes 3.1.2 software (Ronquist and Huelsenbeck 2003). Poorly aligned regions were removed by using the Gblocks server (Castresana 2000). According to their original class, Ulvophyceae clustered together into a single branch, Trebouxiophyceae and Chlorophyceae clustered together into another branch. *Codium fragile* showed a closer relationship with *C. simulans* and *Codium* sp. *'arenicola'* in Ulvophyceae (Figure 1). This complete plastid genome analysis of *C. fragile* helps us better understand the evolutionary process of Chlorophyta.

**Figure 1.**
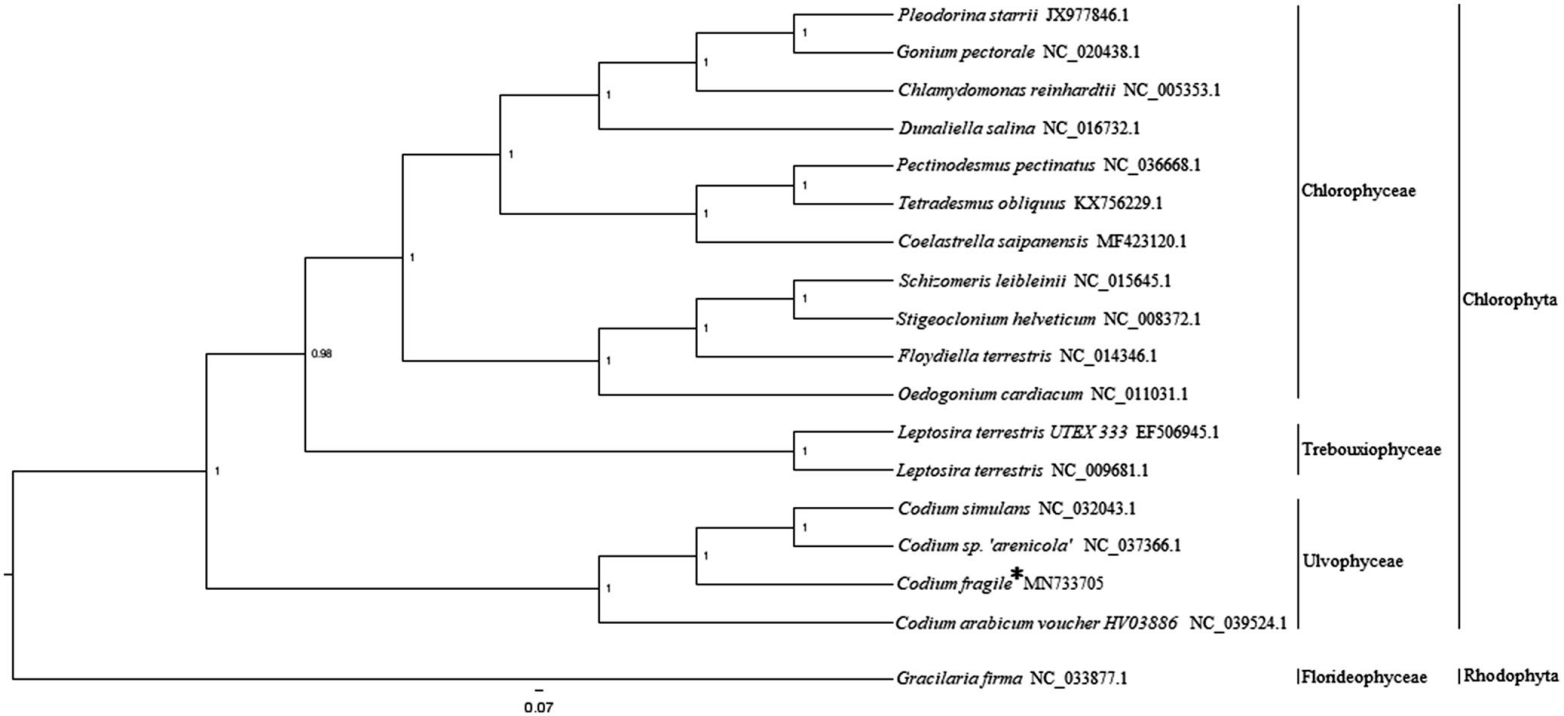
Phylogenetic tree (Bayesian inference) based on complete plastid genomes of Chlorophyta. Support values for each node were calculated from Bayesian posterior probability (BPP). Asterisks following species names indicate newly determined plastid genomes.
